# Cobalt/Bioglass Nanoparticles Enhanced Dermal Regeneration in a 3-Layered Electrospun Scaffold

**DOI:** 10.34172/apb.2024.006

**Published:** 2023-07-22

**Authors:** Zahra Hemmati Dezaki, Kazem Parivar, Vahabodin Goodarzi, Mohamad Reza Nourani

**Affiliations:** ^1^Department of Biology, Science and Research Branch, Islamic Azad University, Tehran, Iran.; ^2^Tissue Engineering and Regenerative Medicine Research Center, Baqiyatallah University of Medical Sciences, Tehran, Iran.

**Keywords:** Cobalt ions, Bioglass nanoparticles, Fibroblasts, Electrospinning technique

## Abstract

**Purpose:**

Due to the multilayered structure of the skin tissue, the architecture of its engineered scaffolds needs to be improved. In the present study, 45s5 bioglass nanoparticles were selected to induce fibroblast proliferation and their protein secretion, although cobalt ions were added to increase their potency.

**Methods:**

A 3-layer scaffold was designed as polyurethane (PU) - polycaprolactone (PCL)/ collagen/nanoparticles-PCL/collagen. The scaffolds examined by scanning electron microscopy (SEM), Fourier transform infrared (FTIR), tensile, surface hydrophilicity and weight loss. Biological tests were performed to assess cell survival, adhesion and the pattern of gene expression.

**Results:**

The mechanical assay showed the highest young modulus for the scaffold with the doped nanoparticles and the water contact angle of this scaffold after chemical crosslinking of collagen was reduced to 52.34±7.7°. In both assessments, the values were statistically compared to other groups. The weight loss of the corresponding scaffold was the highest value of 82.35±4.3 % due to the alkaline effect of metal ions and indicated significant relations in contrast to the scaffold with non-doped particles and bare one (*P* value<0.05). Moreover, better cell expansion, greater cell confluence and a lower degree of toxicity were confirmed. The up-regulation of TGF β1 and VEGF genes introduced this scaffold as a better model for the fibroblasts commitment to a new skin tissue among bare and nondoped scaffold (*P* value<0.05).

**Conclusion:**

The 3-layered scaffold which is loaded with cobalt ions-bonded bioglass nanoparticles, is a better substrate for the culture of the fibroblasts.

## Introduction

 Skin tissue is classified as a tissue with self-renewing and self-repairing abilities and hence, it can regenerate partial wounds. However, the injuries deeper than dermis, are remained as scars^1^ and therefore, some factors and cell sources are needed to encourage their healing via extracellular matrix (ECM) deposition and also, cell growth. Besides, biomaterials must be employed to transfer cells to this tissue locally and preserve them from host immune system.^2^ On the other hand, the limited source of autografts^3^ and the problems related to xeno/allografts,^4^ the development of new strategies are highlighted accordingly. It has been found that if these approaches are designed in accordance with their similarities to dermal matrix, better regeneration will be happened.^5^ Since, human dermal tissue is composed from multiple layers, it would be more beneficial to design a scaffold contained same layers to mimic the architecture of normal skin tissue. While each layer performs a special function, but the more critical point is the integration of these layers with host tissue and each other. These multiple layers may be recruited to improve the mechanical properties of scaffolds, factor release, cell adhesion and even other characters as antibacterial or mucoadhesive potencies. For better mechanical support, polyurethane (PU) as a stretchable polymer same as skin tissue, has been considerably recommended.^6^ A group fabricated a scaffold with 3 layers of polycaprolactone (PCL)-Zein-gum arabic, PCL-*Calendula officinalis *and PCL-Zein-gum Arabic. This structure was used for the delivery of *C. officinalis* in a controlled manner.^7^ Another similar study produced a scaffold with 2 layers including zein film and gentamicin loaded zein layer.^8^ Also, a mat from PCL-cellulose acetate and chitosan-polyethylene oxide, was prepared as a structure with 2 layers to form a dressing with higher mechanical properties.^9^ Among different polymers, collagen as a main component of ECM and particularly, due to its Arg-Gly-Asp (RGD) sequences can attach to integrin receptors on cells.^10^ Another group reported that when collagen was electrospun with hyaluronate, the expression of tissue inhibitor of metalloproteinases by foreskin fibroblasts, was decreased and the related scars were removed. Thus, this factor has been introduced as a tissue inhibitor.^11^ Also, PCL and collagen was evaluated as a blend and the results approved considerably better cellular attachment and proliferation.^12^ On the other hand, collagen must be crosslinked to develop a stable scaffold. If collagens are washed out easily, its mechanical behavior and cell anchoring will be decreased. Thus, for a higher mechanical strength and stretching, natural polymers are always blended with synthetic types.^13^ It had been approved that PCL has the strength with 2.5 times of normal human dermal tissue. Regarding to this, PCL was blended with collagen by a group to increase its scaffold strength.^14^ A report demonstrated normal matrix reorganization, angiogenesis, faster wound closure and hair follicle production by using electrospun PCL/collagen in the culture of J2 mouse fibroblasts.^15^ L-929 fibroblast cell line which is originated from mice, is used as a standard cell line for dermal tissue engineering.^16,17^ It had been approved by a group that the extracellular vesicles which are produced by L-929 fibroblasts, caused scarless wound regeneration, collagen synthesis and higher proliferation of endothelial cells.^16^ It should be added that for some dermal injuries, the factors including scaffold and cell source alone are not sufficient. With the development of new sciences as nano-technology, several matters have been created and introduced them for skin tissue engineering. 45s5 bioglass nanoparticles have the ability to remodel the dermal damages with large scale.^18^ Also, it had been approved that these nanoparticles increased cell attachment and proliferation when are applied on rat skin scars.^19^ Their antibacterial impact would be another facility of these nanoparticles. In a study, the composite scaffold with bioglass, led into a better dermal repairing and also, the formation of mature vessels.^20^ Moreover, another survey was done to investigate the higher secretion of growth factors as vascular endothelial growth factor (VEGF) by fibroblasts.^21,22^ A related study showed that these particles activate Wnt/β-catenin pathway enhancing the upregulation of insulin like growth factor 1 (IGF1) and transforming growth factor beta (TGFβ). These genes are involved angiogenesis.^23^ Although, the corresponding effects of bioglass particles could be promoted by the substitution with other metals. Among the various nanoparticles, metal groups have attracted many applications in dermal tissue engineering due to their higher biological activities. In an investigation where ZnO, Fe3O4 and Au nanoparticles were added to the poly (lactic acid)/chitosan scaffold, greater dermal full thickness wound healing was occurred, despite their different mechanical properties and biological activities.^24^ Another study printed polycaprolactone-block-poly(1,3-propylene succinate) (PCL-PPSu) and doped the scaffold with silver nanoparticles. The composite scaffold showed higher degradation and lower bacterial adhesion.^25^ Polydopamine scaffold impregnated with TiO2 nanoparticles, promoted cell adhesion, proliferation and migration in compared to the group without these nanoparticles.^26^ Cerium ions were one of metals which was used for the bioglass doping and the related founds confirmed a higher cell attachment and expansion.^27^ Another report is about borate ions that increased the healing of diabetic wounds even 10 times faster.^28^ Also, it was illustrated that strontium ions triggered the dermal healing capability of the bioglass.^29^ Among these metals, cobalt ions have been discussed to possess a considerable role on the angiogenesis role of the bioglass. The related mechanism is that these ions can mimic hypoxia condition and thus, activate the formation of blood vessels. It should be added that cobalt ions must be released to apply this impact.^30^

 In this study, an electrospun scaffold with 3 layers was prepared and the layers were PU, PCL-collagen-cobalt doped bioglass and PCL-collagen. The bioglass nanoparticles with the formulation of 45s5 were doped by cobalt ions to result better healing and antibacterial properties. These nanoparticles were loaded in the middle layer of PCL-collagen and the third layer of PU was only added to increase the scaffold mechanical functions. EDC/NHS compounds were utilized for the crosslinking of collagen fibers. The cell type was L-929 fibroblasts due to its considerable ability in dermal tissue engineering. The related assays of cell attachment and survival were carried out in the following and their gene expression profile was evaluated, too.

## Materials and Methods

###  Scaffold preparation 

 All scaffold groups contained 3 layers, was fabricated with the following steps. First of all, the external layer of polyurethane (thermoplastic PU, Desmopan, cat. no DP8785A) was prepared by dissolving of PU at the concentration of 7%. The solvent contained tetrahydrofuran (THF, Merck, cat. no 108114) and dimethylformamide (DMF, Merck, cat. no 103053) at the ratio of 75/25. It is worth to be noted that the electrospinning process for each layer was 2.5 hr. Also, the related parameters of each were optimized to result beadles fibers. Afterwards, the middle layer was designed to contain polycaprolactone (PCL, 70 kDa, Sigma, cat. no 440744) and collagen (type I, MedZist). The scaffolds were classified to 2 groups by containing either cobalt-doped bioglass 45S5 nanoparticles (MedZist) or non-doped one (MedZist). For this layer, the PCL solution was produced at the concentration of 5% in a solvent of hexafluoro-2- propanol (HFIP, Sigma, cat. no 99 920-66-1). Moreover, the dissolved collagen (2%) in HFIP was added to the PCL solution when both solutions were homogenous. The ratio of PCL and collagen was considered as 80/20, respectively. For the development of a composite form of this layer, the corresponding nanoparticles was added at 1%. At the end, this layer was electrospun similar to the first one for 2.5 hours. The internal layer was fabricated same as the middle one, although in the absence of the nanoparticles. The stirring step for all solutions was performed for 8 hours and at room temperature (RT). Then, the solutions were ready to electrospun (Nanoazma, Iran). For the crosslinking procedure, N-Hydroxysuccinimide (NHS, Sigma, cat. no 6066-82-6) and 1-Ethyl-3-(3-dimethylaminopropyl) carbodiimide (EDC, Sigma, cat. no 25952-53-8) were recruited at the concentrations of 2 and 3 mg/mL in ethanol (Merck, cat. no 100990).^31^ The scaffold samples including the control and test groups (without and with nanoparticles respectively) were incubated at the temperature of lower than 10 °C for 3 hours and then, 1 hour at ambient temperature for the following assessments.

###  Scanning electron microscopy (SEM) examinations 

 SEM (Seron Technologies - AIS2100 model, Gyeonggi-do, Korea) method was utilized to evaluate the diameter and distribution of the generated fibers by electrospinning method. However, before this morphological examination, gold ions were coated on the scaffolds by using an ion sputter (JFC-1100, JEOL, Japan) for 15 min and under vacuum pressure. Herein, there were 2 control groups including a 2-layer scaffold without the nanoparticles (PU and PCL-collagen) and 2-layer scaffold with the nanoparticles (PU and PCL-collagen-nanoparticles). The experimental groups were the 3-layer scaffold types with and without the nanoparticles. Also, they were examined as crosslinked and non-crosslinked types. For the measurement of fiber diameter, ImageJ software was employed in the following and the number of the fibers for each scaffold group employed to obtain the fiber values, was 25.

###  FTIR spectroscopies 

 The chemical characterization of the polymers and nanoparticles within the corresponding scaffolds (control and experimental groups) is necessary. For this approach, both scaffold types were studied by using Fourier transform infra-red (FTIR, ATR-FTIR Thermo Nicolet model: NEXUS 670, USA). All spectra within the range of 500 – 4500 cm^-1^, were normalized by KBr pellet. Moreover, the values of the resolution and scan rate were 4 cm^-1^ and 120 mV/min, respectively.

###  Tensile characterizations 

 The elongation potential of the constructed scaffolds could influence on their dermal regeneration abilities. Thus, for this assay, the scaffolds were cut as a rectangular with the dimension of 5 mm × 30 mm and then, the samples were fixed in the tensile apparatus (SANTAM universal tensile testing device, SPM20, Iran). The measurement was repeated for 3 times and the curves with its Excel file was extracted for the following analysis. This mechanical assay was carried out by the velocity rate of 1 mm/min and the fatigue limit of 0.5 kN. The resultant curve had a linear region in the first elastic part and its slope was considered as the value of young modulus.

###  Water-contact angle measurements 

 All fabricated scaffolds of the non-composite and both composite types including with and without cobalt ions were evaluated for their surface hydrophilicity. This property was measured by a G10 Kruss contact angle goniometer. After fixing the scaffold samples on the stage, the contact angle between a sessile water drop and the scaffold was recorded in the following. The reported contact angle in this study is related to 10^th^ second.^32^

###  Weight loss analysis 

 The established scaffolds including the control and test groups were investigated about their degradation rate. First of all, the dried weight of the sterilized scaffolds was measured and then, they were incubated in the media of sterile injection water. The samples were removed after 7, 14, 21 and 28 days from the incubator and washed several times by phosphate buffered saline (PBS). For their complete drying, the scaffolds were transferred to an oven (Memmert, Type UNB 400, Schwabach, Germany) for 30 minutes at 60 °C and at last, they were weighted. The weight loss (%) was calculated by using the below equation^33^:

 Weight loss (%) = [W0 - Wt / W0] × 100

 Herein, W0 presents the dried weight of the scaffolds at zero-time point and accordingly, Wt indicates the dried weight after t time.

###  Cell culture

 L-929 fibroblasts cell line (Pasture Institute, Iran) was selected as a cell source and expanded for 2 weeks to have enough cells for the evaluations. The scaffolds were washed several times by using PBS (Thermofisher, cat. no 003002) and then, they were cut at a predetermined size and sterilized in the following. The sterilization process was carried out by the employment of filtered 70% ethanol for 1 hour and then, an extra process of UV irradiation for 20 minutes. The cells were peeled by trypsin (Gibco, cat. no 15090046) and counted using a neobar slide. About 15 × 10^3^ cells were seeded on 1 × 1 cm^2^ of the scaffolds. The cell culture media of Dulbecco’s modified Eagle’s medium (DMEM, Thermofisher, cat. no 11965092) high glucose with 10% of fetal bovine serum (FBS, Thermofisher, cat. no 10082147) was added to the scaffolds and stored in an incubator with the temperature of 37 °C and 5% CO2.

###  DAPI and SEM assessments 

 To insure about cell adhesion, 4′, 6-diamidino-2-phenylin-dole (DAPI, Sigma, 5 µg/mL, cat. no 28718-90-3) and SEM methods were employed. For DAPI staining, the scaffold samples at 1, 3 and 7 days were treated with glutaraldehyde (Sigma, 2.5%, cat. no 111-30-8) for 45 minutes and at RT. Then, the groups were washed by PBS and DAPI solution was added for another 20 min. After all, the DAPI reagent was washed and replaced by PBS. Their images were taken by fluorescence microscopy (Nikon, Eclipse TE2000-S, Japan). Although, tissue culture polystyrene (TCPS) group as the control group was studied too. On the other hand, SEM technique was used to detect cell morphology on the scaffolds. Again, after 14 days of the cell seeding, both the control and test scaffolds were incubated in glutaraldehyde (2.5%) for 1 hour at RT. After that, the scaffolds were dehydrated by using the ethanol solutions with the dilutions of 50%, 60%, 70%, 80%, 90% and 100%. The time incubation for each step was 20 minutes. The scaffold specimens were studied by SEM after gold sputtering as same as the method of the fiber diameter measurements in the before section.

###  MTT assay 

 The bioactivity of the fabricated scaffolds was evaluated by using L-929 fibroblast cell line. The biotoxicity of the scaffolds were studied by using 3-[4, 5-dimethylthiazol-2-yl]-2, 5 diphenyl tetrazolium bromide (MTT, Sigma, cat. no 11465007001). For this assessment, the MTT compound with the dilution of 0.1 mg/mL was prepared in DMEM without FBS and added to the cell wells. After 3.5-4 hours of the incubation in the dark, dimethyl sulfoxide (DMSO, Merck, cat. no 102952) was added to dissolve the reduced form of MTT (purple formazan). Herein, TCPS was defined as the control group to measure cell viability values. Their optical densities (OD) were read at 570 nm and the cell viability (%) values were calculated by using the below equation:

 Cell viability (%) = Optical density (OD) experiment /control group / OD of TCPS × 100

###  Real-time polymerase chain reaction (PCR) technique

 Herein, real-time PCR seems to be very applicable to detect the changes about the gene expression of the seeded cell on the scaffolds. The assay was performed at 3rd and 7th days of cell culture. For this aim, the total RNA of the cells were extracted by TRIzol reagent (Sigma, cat. no T9424) and their associated cDNA was synthesized accordance with an optimum protocol. For the cDNA production, M-MuLV reverse transcriptase (RT) and Random Hexamer were bought from Fermentas (cat. no 28025013 and N8080127, respectively). For the real-time PCR reactions (Rotor-gene Q software, Corbett), 0.5 µL of cDNA was used for each test sample and the parameters of temperature and time, were set as 94 °C for 3 minutes for the annealing temperature, the conditions of 35 cycles (94 °C for 30 seconds, 62 °C for 45 seconds, 72 °C for 45 seconds) and the extension time of 7-10 minutes at 72 °C. The relative gene expression values were obtained by the comparative ∆∆Ct method.^34^ The employed real-time PCR master mix was afforded from Fermentas (cat. no 4309155). Moreover, the primer sequences were collected in Table 1. Glyceraldehyde-3-phosphate dehydrogenase (GAPDH) as the housekeeping control and the other 2 genes of VEGF-A and TGF-β1 were the experimental genes.

**Table 1:** The primer sequences of GAPDH, TGF β1 and VEGF genes which was employed in the present study

**Gene name**	**Sequences **	**Tm**
GAPDH-F	CAAGTTCAACGGCACAGTCA	57.30
GAPDH-R	CCCCATTTGATGTTAGCGGG	59.35
VEGF-F	GCAATGATGAAGCCCTGGAG	59.35
VEGF-R	CCTATGTGCTGGCTTTGGTG	59.35
TGFβ-F	ATGACATGAACCGACCCTTC	57.30
TGFβ-R	ACTTCCAACCCAGGTCCTTC	57.30

###  Statistical considerations 

 Sigma-plot software was recruited for all statistical calculations. Moreover, the related assay of student’s t-test was chosen to find out the difference between 2 groups of the control and test types. Regarding to this, the *P *values of equal or lower than 0.05, was considered as significant differences. On contrast, the higher value (*P* > 0.05) was reported as insignificant relation. In this study, all assays were done as triplicate and thus, the values were indicated as mean ± standard error.

## Results and Discussion

###  Fiber diameter and distribution by SEM characterization

**Figure 1:**
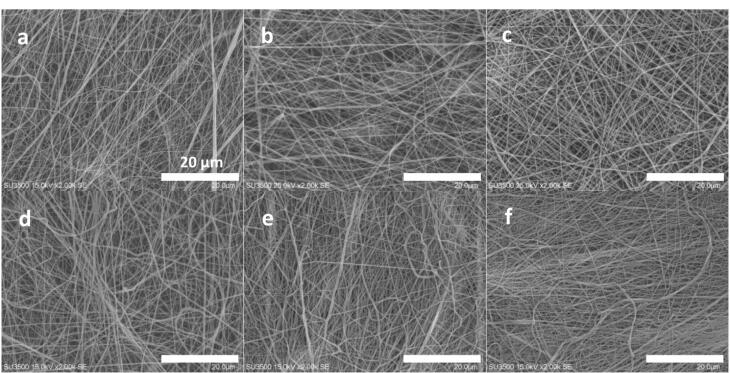
SEM fiber morphology of the 2-layer bare scaffold before crosslinking (a), the 2-layer 45s5 bioglass composite scaffold before crosslinking (b), the 2-layer 45s5 bioglass composite scaffold with crosslinking (c), the 2-layer cobalt doped-45s5 bioglass composite scaffold before crosslinking (d), the 3-layer cobalt doped-45s5 bioglass composite scaffold prior to crosslinking (e) and the 3-layer cobalt doped-45s5 bioglass composite scaffold after crosslinking (f)

 The developed scaffolds including the bare and composite types were evaluated about their fiber diameter and morphology. The results are indicated in Figure 1. Due to the multi-layer design of the scaffolds, the both formats with 2- and 3-layer types were reported here. The all related electrospinning parameters such as the tip-collector distance (cm), applied voltage (kV), debi (mL/h) and the rotation speed of the collector (rpm) were optimized and reported in Table 2. The criteria for the optimization of these parameters was depended on the beadless morphology of the produced nanofibers. The distance and collector rotation rate were set stable to get similar fiber numbers for the all layers. It is clear that the applied voltage was higher for the PU solution as a function of its higher surface tension. Additionally, the less flow rate of the corresponding polymer confirms again its lower electrostatic charges. The related figure to the bare scaffold which was considered as the control group, had 2 layers including the PCL/collagen (inward part) and PU (external part). Its fiber diameter was resulted as 162.55 ± 37 nm, but the fiber diameter was enhanced significantly when the 45s5 bioglass nanoparticles was added. The value was 205.52 ± 83 nm confirming the reduction of the electrospun solution conductivity due to the insulating character of the glass nanoparticles.^35,36^ This difference is statistically distinguishable (*P* < 0.05). The crosslinking process of the collagen polymers within the scaffolds were carried out by using EDC/NHS which have been known as zero-length crosslinkers.^37^ Regarding to this, the fiber diameter of the latter scaffold after its crosslinking was calculated as 198.12 ± 75 nm that is no considerable difference with the fiber diameter of this scaffold before the crosslinking (*P* > 0.05). Additionally, the standard deviation (SD) values may be informative about the fiber dimeter homogeneity. For the control group, the SD value is lower, however, the value is higher in the composite groups. In our knowledge, this point is related to this fact that some fibers can catch the nanoparticles and the others remained non-occupied. Therefore, their diameter values were fluctuated and strictly depended on the presence or absence of the nanoparticles. In other words, these nanoparticles manipulate the solution conductivity locally and finally, the developed fibers possess different diameters. The composite scaffold with the cobalt-doped version of the bioglass nanoparticles are discussed as the groups with 2 and 3 layers. The fiber diameter of 2-layer form of this non-crosslinked scaffold was obtained as 206.14 ± 66 nm demonstrating no significant difference in compared to its counterpart with the non-doped nanoparticles (*P* > 0.05). However, the fiber diameter of the 3-layer structure before and after the chemical crosslinking was not changed (*P* > 0.05). It should be added that the diameter values were reduced to 197.86 ± 84 and 202.84 ± 67 nm, respectively for the non-crosslinked and crosslinked types. In this manner, the fiber diameter was statistically kept stable after the addition of the cobalt ions. This phenomenon could be justified that in contrast to other studies, the presence of cobalt ions could not increase the electrical conductivity of the solution contained these polymers and solvents.^38-40^ It is also clear that in the all scaffold groups, there are many rooms between the fibers and these channels within the scaffolds are known as pores. Similar to the physiological conditions, the circulatory system transports nutrients, drugs, hormones and gases throughout the body. Thereby, the scaffold used to regenerate cell defects, must deliver and wash out various compounds through its pores in the absence of vessels. Proper porosity is essential for the transfer of water, nutrients and cellular artifacts and also, culture cells can easily communicate with each other or even control their migratory behavior.^41^ On the other hand, the porosity must be connected for better material transfer throughout the area of a scaffold.^42^ The presence of pores would help control autocrine and endocrine signaling between cells.^43^ It should be noted that these pores should be uniform and their size can be controllable to tune tissue requirements.^44^ As it is resulted here, the all developed fibers had nanoscale diameters that is essential for providing higher surface area for cell adhesion compared to TCPS.^45^

**Table 2:** The electrospinning parameters which were used to produce beadless fibers of PU, PCL/collagen/bioglass, PCL/collagen/cobalt doped bioglass and PCL/collagen scaffolds

**Electrospinning parameters/Scaffold groups**	**Voltage (kV)**	**Infusion rate (mL/h)**	**Collector rotation speed (rpm)**	**Distance (cm)**
PU	16.5	0.2	300	18
PCL/collagen/bioglass	15	0.3	300	18
PCL/collagen/cobalt doped bioglass	15	0.3	300	18
PCL/collagen	15	0.2	300	18

###  Characterization of chemical groups by FTIR spectroscopy 

**Figure 2:**
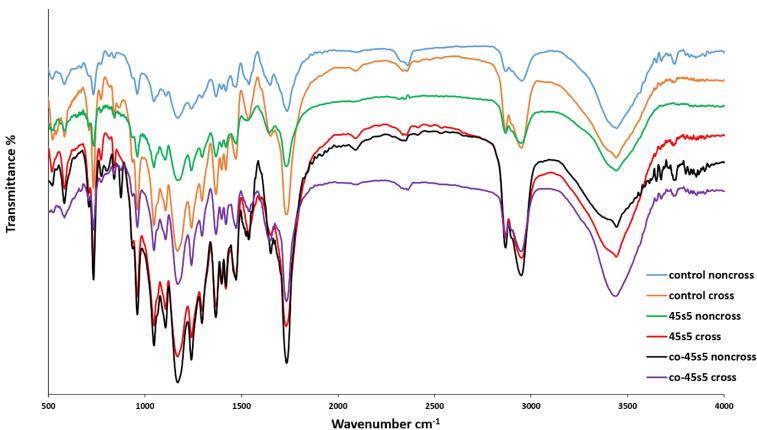
The FTIR spectroscopies of the 2-layer bare, 3-layer 45s5 bioglass composite and cobalt doped-45s5 bioglass composite scaffolds with and without crosslinking with EDC/NHS

 The scaffolds including the bare and composite types were explored for their chemical functional groups (Figure 2). Also, since the chemical crosslinking can alter the chemical properties of the materials, this assay was carried out for the both crosslinking and non-crosslinking forms. As it is apparent, the carbonyl and hydroxyl functional groups are exposed a sharp spectrum at 1726^46^ and 3430 cm^-1^,^47^ respectively. These chemical groups are belonged to the PU component of the all scaffolds contained this polymer. Thus, the specific peaks of PU had been exposed by the all groups due to this material was one of the major constituents of the prepared scaffolds. Another bonds at 2922^48^ and 2845^49^ cm^-1^ as a function of CH_2_ and CH stretching, are detectable for the PU polymer. The isocyanate functional groups made a peak at 2312 cm^-1^.^50^ Although, its intensity had been influenced by the nanoparticles due to the close interactions between the negative charge atom of bioglass and the nitrogen atoms of isocyanate. The stretching of N-H and C = O within the PU chemical structure produced other peaks at 1570 and 1608 cm^-1^.^50^ It must be added the intensity of these mentioned peaks at above, was enhanced after the scaffold crosslinking by EDC/NHS.^51^ It could be justified that the crosslinking of collagen limits the chains of this protein and then, their bonds with other matters as PU will be reduced. The CH_2_ groups of the PU and PCL polymers developed some peaks at the region of 750-700 cm^-1^ due to their rocking modes.^52^ Similarly, these peaks showed higher intensities with the scaffolds which were crosslinked. The 2 clear peaks which was attributed to the C-O-C stretching frequency in PCL, were resulted for the all scaffolds.^53^ Amide type I and II within the chemical structure of collagen created 2 peaks at 1657 (C = O) and 1553 (N-H) cm^-1^ those are same about PU and collagen.^54^ These peaks have been amplified after the crosslinking except for the scaffold with the doped nanoparticles. The presence of cobalt ions could be characterized here by their chemical connections with the collagen and PU chains. Also, amide type III has a particular peak at 1237 cm^-1^ due to the stretching of C-N bonds.^55^ 45s5 bioglass nanoparticles caused some bonds between 1024–500 cm^-1^ as the representative of Si-O-Si groups due to their non-asymmetric and asymmetric vibrations.^56^ The several peaks of this area could be concerned to calcite^57^ within the bioglass structure. The carbonate groups of the bioglass appeared a peak at 1450^58^ cm^-1^ with a higher intensity after the cross-linking but not for the composite scaffold with the substituted bioglass by cobalt. The intensities of the associated peaks to the non-bridging oxygen at 721 cm^-1^, were increased due to cobalt ions within the cobalt-introduced bioglass PU-PCL/collagen/nanoparticles-PCL-collagen scaffold. Moreover, the intensities of the all specific bonds of Si-O-Si groups in the bioglass nanoparticles are triggered as a function of breaking of these bonds by cobalt ions.^59^

###  Mechanical properties by tensile method 

**Figure 3:**
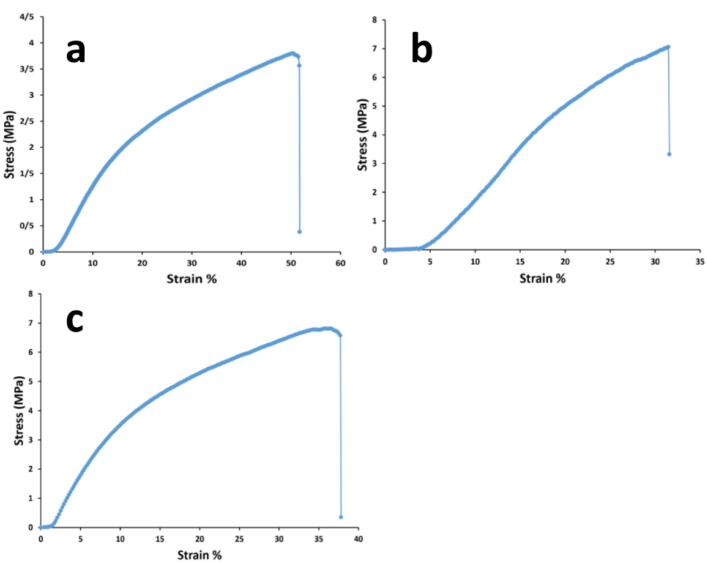
The mechanical tensile curves of the 2-layer bare, 3-layer 45s5 bioglass composite and cobalt doped-45s5 bioglass composite scaffolds after their crosslinking with EDC/NHS

 A scaffold with mechanically matched properties must be considered for their designing. If host tissue and scaffolds are not mechanically integrated with each other, the implantation will be failed.^60^ Although, scaffold mechanical attributes are reduced during tissue regeneration due to its replacement with the new organized tissue. Herein, due to the thermoplastic property of PU, the significant elastic performance of the prepared scaffolds were anticipated.^61^ On the other hand, PCL is usually added to collagen to increase its poor tensile strength.^14^ Additionally, the collagen crosslinking by using EDC and NHS, has been suggested mostly to increase its mechanical functions, as well as its fibrillary alignment during healing.^62^ The results of mechanical tensile assay in this section contributes to the scaffolds including the bare and composite types and were gathered in Figure 3 and Table 3. It should be noted that the all scaffolds possessed 3 layers and were chemically crosslinked in accordance with the mentioned protocol in methods. The all values were obtained by the evaluation of triplicate samples and reported as average value ± SD. Herein, the mean tensile strengths of the bare and composite with the cobalt doped nanoparticles have insignificant relations (*P* > 0.05) as 6.32 ± 0.82 and 6.41 ± 0.70 MPa, respectively. In spite of this, the correlated value of the composite scaffold with the non-substituted nanoparticles is 8.56 ± 2.13 MPa (*P* < 0.05). In accordance with before surveys, the corresponding mechanical resistance is increased after the addition of bioglass nanoparticles.^63^ This result could be concerned to nanoparticle localizations among polymer fibers leading to higher stress values. Along with this, its young modulus is the ultra-low value as 1.36 ± 0.07 MPa signifying higher flexibility with this scaffold.^64^ Especially, when the elongation percentage of this scaffold is the highest value (*P* < 0.05) as 59.47 ± 10.31 %, its better stretching in compared to other groups is interested. The young modulus quantities of the bare and co-bonded bioglass composite scaffolds were 3.32 ± 1.90 and 6.75 ± 1.48 MPa, respectively. In a competitive consideration between the scaffolds, the latter composite scaffold due to its highest modulus could be introduced as a most tough substrate (*P* < 0.05). The data confirmed that the scaffold can endure any high loaded force with the lower elongation change and hence, the scaffold is relatively intact at higher forces compared to the composite scaffold with the non-doped nanoparticles. Also, this high young modulus value approved that this scaffold is the stiffer type among the scaffolds.^65^ It should be added that a stiff substrate is wanted for cell culture approaches to provide attachment sites for adherent cells as fibroblasts. A similar study earned a same result about the higher mechanical stability after the addition of strontium ions to bioglass nanoparticles.^66^ Also, after the employment of silver ions as a dopant of bioglass, again higher value of young modulus was resulted.^67^ The corresponding composite scaffold indicated 55.51 ± 7.21% as its ultimate strain value that has no significant distinguish with the bare scaffold with the value of 55.62 ± 7.36% (*P* > 0.05). In this manner, when bioglass nanoparticles are bonded with metal ions as cobalt, their interactions with polymer chains are reduced and the polymers become free and can behave similar to the bare scaffold.

**Table 3:** The tensile parameter amounts of PCL/collagen/bioglass, PCL/collagen/cobalt doped bioglass and PCL/collagen scaffolds

**Values/scaffold groups**	**Max Stress (MPa)**	**Max Strain (%)**	**Young Modulus (MPa)**
Bare scaffold	6.32 ± 0.82	55.62 ± 7.36	3.32 ± 1.90
45s5 bioglass scaffold	8.56 ± 2.13	59.47 ± 10.31	1.36 ± 0.07
Co-doped scaffold	6.41 ± 0.70	55.51 ± 7.21	6.75 ± 1.48

###  Surface hydrophilicity by water-contact angle examination

**Figure 4:**
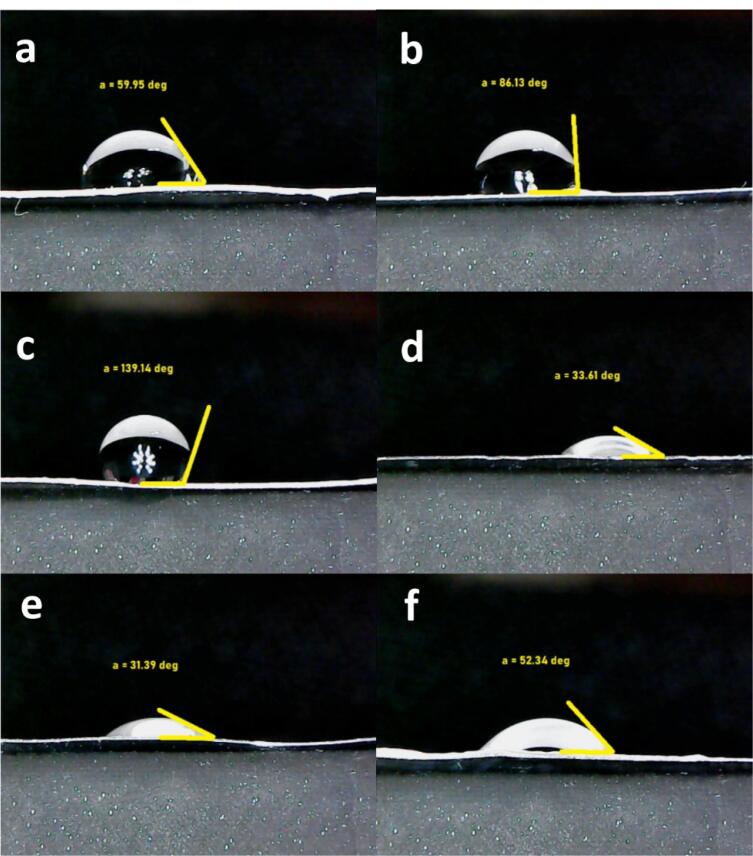
The water contact angle values of the 2-layer bare scaffold before crosslinking (a), the 2-layer bare scaffold after crosslinking (b), the 3-layer 45s5 bioglass composite scaffold prior to crosslinking (c), the 3-layer 45s5 bioglass composite scaffold with crosslinking (d), the 3-layer cobalt doped-45s5 bioglass composite scaffold before crosslinking (e), the 3-layer cobalt doped-45s5 bioglass composite scaffold after crosslinking (f)

 Optimized surface wettability is needful for the communications between cells and substrates^68^ and with as much as its high values, cell attachments would be encouraged more.^69^ The cells that had been selected for this study as introduced in before sections, were L-929 fibroblast cells. These cells adhere to surfaces hardly and they are not separated easily even with several washing times.^70^ On the other hand, the corresponding cell growth critically depends on their adherence ability. As noted for the development of an appropriate dermal implant, cell attachment seems necessary. By considering this fact, the scaffolds surface was examined by their contact angle with water molecules and shown in Figure 4. In accordance with before reports, if the water contact angle of a substrate is lower than 90°, the scaffold could be employed as a hydrophilic membrane.^71^ This contact angle scale is enough to develop a surface which cells can attach on it by overtaking water surface tension.^72^ At a short glance, the all angle values are lower than 90°, except the composite scaffold with the non-doped nanoparticles. The contact angle with water was statistically significant (*P* < 0.05) between the crosslinked and non-crosslinked scaffolds. The results approved the considerable chemical impact of the crosslinking by EDC/NHS. Moreover, the angle values are higher after the crosslinking and as a whole, this process converts the substrates to weaker hydrophilic types. This result may be related to the gathering collagen fibers by the chemical crosslinking and PCL chains become free to apply its hydrophobic nature.^73^ This event was resulted similarly for the composite scaffold with the bioglass nanoparticles doped by cobalt ions and the bare scaffold. Their values were respectively changed from 59.95 ± 4.7° to 86.13 ± 6.5° and 31.39 ± 2.8° to 52.34 ± 7.7°. The values of the prior and after the crosslinking, have significant differences (*P* < 0.05) confirming the qualified chemical crosslinking by using EDC and NHS. In conflict with this data, the correlated hydrophilic level of the composite scaffold with the non-doped nanoparticles was altered from 139.14 ± 5.4° to 33.61 ± 3.5°. This result may be associated to the absence of cobalt ions with 45s5 bioglass nanoparticles. Therefore, the charges of the bioglass is not limited by cobalt ions and the surface wettability would be enhanced in the presence of these ions. In other meaning, the hydrophilic property of the bioglass nanoparticles is capable to eliminate the hydrophobic role of EDC/NHS crosslinking. The contact angle of the co-bioglass scaffold is the lowest value in compared to the control and non-substituted composite scaffold (*P* < 0.05). In this condition, the interactions of water with the doped nanoparticles are established through van der Waals bonds rather than electrostatic. In contrast, the bare and non-bonded bioglass scaffolds do this process by their hydrophobic and ionic interactions, respectively (Table 4). By considering the value of contact angles, the non-doped composite scaffold possesses the lowest value even lower than 45°^74^ and thus, more desired cell interactions would be occurred.

**Table 4:** The properties of 3-layer co-doped scaffold as the optimized group for the regenerative approaches of skin tissue

**Parameter **	**Fiber diameter ** **(nm)**	**Young modulus (MPa)**	**Water contact angle (⸰)**	**Weight loss after 28 days (%)**	**Cell viability after 14 days (%)**	**Expression of VEGF and TGFβ1**
Values	202.84 ± 67	6.75 ± 1.48	52.34 ± 7.7	82.35 ± 4.3	137.07 ± 8	79.57 ± 4 and 109.13 ± 5

###  Degradation rate of prepared scaffolds 

**Figure 5:**
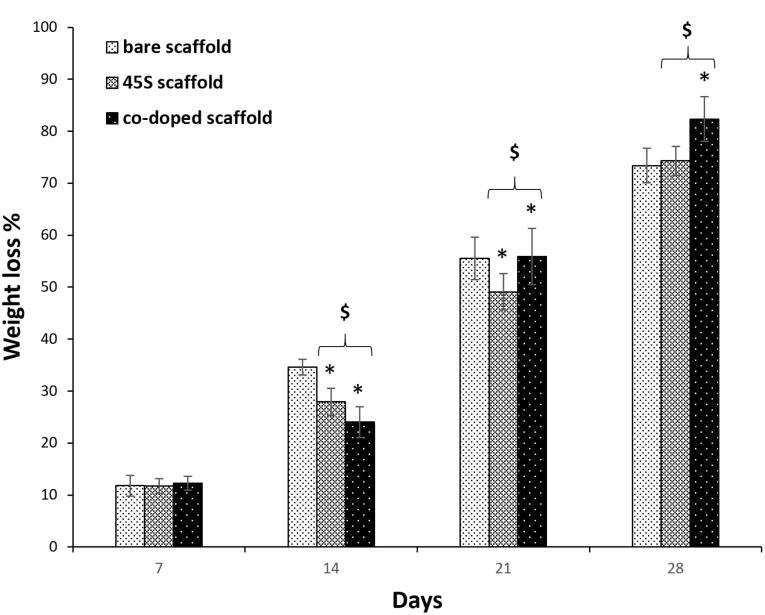
The weight loss of the 2-layer bare, 3-layer 45s5 bioglass composite and cobalt doped-45s5 bioglass composite scaffolds after their crosslinked with EDC/NHS. The star signs indicate the difference between the bare and co-doped bioglass scaffolds and the dollar signs indicate difference between the non-doped and co-doped bioglass scaffolds. Two and three signs show the p-values lower than 0.005 and 0.0005, respectively

 For all kinds of tissues, the control of scaffold biodegradation is interested to optimize between its degradation and the production of a new tissue. Also, it is considered important that when a scaffold is absorbed by the surrounding tissues during the degradation process, it will not make any toxic responses.^75^ On the other hand, if a scaffold is capable to degrade, there is no need to remove them by surgeries. Herein, the polymer types of the scaffolds were thermoplastic PU, PCL and collagen. It has been approved that the employed PU degrades as a result of hydrolyzing reactions on its diols fragments including PCL and carboxybetaine.^76^ However, this polymer has been well-known as a slow weight losing material and only about 11% of this polymer was degraded after 30 days.^77^ PCL which had a molecular weight of 80 kDa in this research, can be hydrolyzed due to its aliphatic ester bonds. However, the pristine PCL losses about 18% of its weight after 30 days and therefore, it has a slow degradation.^78^ The last polymer is collagen which was crosslinked by using EDC/NHS. However, in spite of this, it was expected its total degradation after 7 days accordance with before studies.^31^ The results of the prepared scaffolds were indicated in Figure 5. It is apparent that the addition of ceramics as bioglass, could decrease the degradation rate of the polymers significantly after 14 and 21 days (*P* < 0.05).^79^ Its relation after 28 days was not statistically significant compared to the control group (*P* > 0.05). Although, by the incorporation of cobalt ions within the bioglass structure, the degradation was accelerated with the significant relations against the naked and non-doped composite scaffolds. The result was in agreement with a study that concluded the degradation of PCL was increased in the presence of doped particles with cobalt ions.^80^ As a conclusion, it is possible to adjust the scaffold degradation rate by these nanoparticles. This high degradation after applying higher pH, makes more releasing of the loaded cobalt ions.^59^ Alkaline condition has been confirmed that makes the higher degradation of PCL^81^ and the same fate is occurred for PU.^82^ The delivery of cobalt ions is necessary to their antibacterial and also, healing impacts. However, the release amount must be optimized by choosing an appropriate cobalt concentration to inhibit their possible toxicity.

###  Cellular adhesion on the prepared scaffolds by SEM and DAPI assessments 

**Figure 6:**
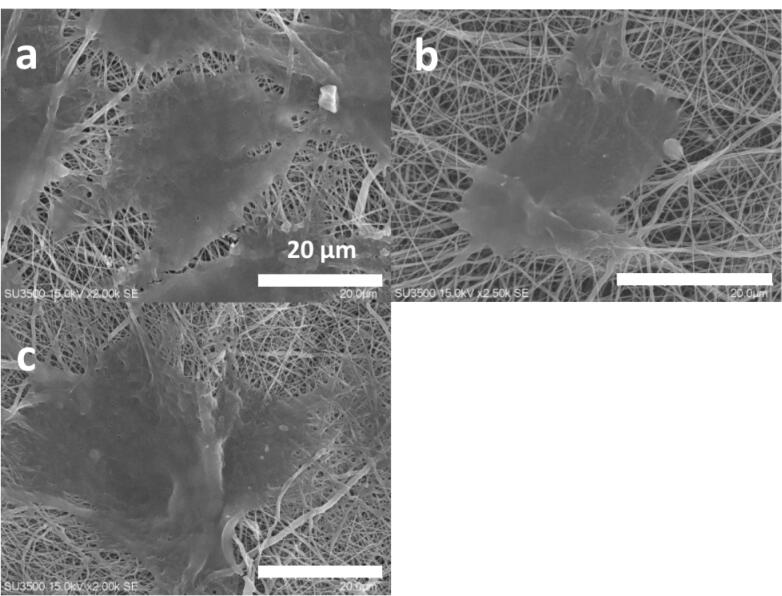
The SEM cell attachment studies of the 2-layer bare (a), the 3-layer 45s5 bioglass composite (b) and the cobalt doped-45s5 bioglass composite (c) scaffolds after their crosslinking with EDC/NHS

**Figure 7:**
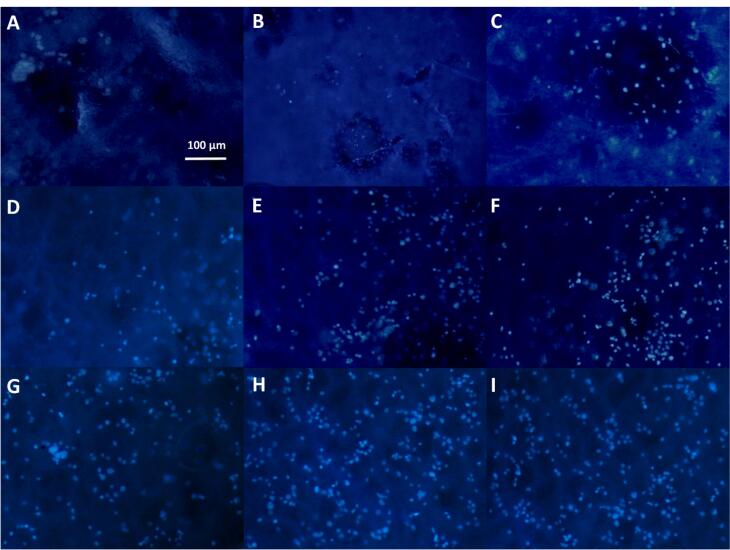
The DAPI staining of the 2-layer bare after 1 day (A), the 3-layer 45s5 bioglass composite after 1 day (B) and the cobalt doped-45s5 bioglass composite after 1 day (C), the 2-layer bare after 3 day (D), the 3-layer 45s5 bioglass composite after 3 day (E) and the cobalt doped-45s5 bioglass composite after 3 day (F), the 2-layer bare after 7 day (G), the 3-layer 45s5 bioglass composite after 7day (H) and the cobalt doped-45s5 bioglass composite after 7 day (I)

 Cells spreading and their extension on substrates are critical for tissue engineering aims. Herein, Figure 6 depicts the morphology of L-929 fibroblast cells on the non-composite and composite scaffolds. The composite groups had 3 layers which contained the nanoparticles within its middle layer. However, one composite type was loaded by the non-doped bioglass nanoparticles and the other by the cobalt-substituted type. L-929 fibroblast cells have a spindle form with a size of about 45 µm.^83^ Surprisingly, their diameter was larger after their culturing on the composite scaffold with the coupled form of 45s5 bioglass nanoparticles. However, the cells had lower spreading ability on the control and the composite scaffold with the non-doped type of the nanoparticles. Even, the morphology was more non-expansion on the latter scaffold compared to the bare group. Regarding to this, it had been approved that these cells show a smaller size when they are cultured on the topographies with the special chemical properties^84^ and hence, their smaller expansion may be occurred depending on surface chemical properties. While, it should be added that nanofibrous substrates provide better cell adhesion and spreading due to their higher surface area.^85^ It seems that the cells on the scaffold with 45s5 bioglass nanoparticles are retracted to some extent and their filopodia are removed in contrast to the control and cobalt-doped composite scaffold. The corresponding shrinkage could be related to the stiffness of the scaffold surface^86^ as demonstrated in before section by the higher young modulus of this composite scaffold type. This event leads into the weaker healing potency of cells and probably enforces scar creation.^87^ Additionally, the SEM observations confirmed a higher cell density on the control and cobalt-bonded scaffold in compared to the composite scaffold with the non-substituted nanoparticles. Although, better cell spreading on the composite scaffold is apparent compared to the control group. This correlation between cobalt ions and cell expansion had been approved by other studies.^88^ Besides, it should be added that the all scaffold groups due to the presence of collagen within their structures, must support cell adhesion. Collagen increases cell attachment as a function of its RGD sequences within its amino acids. In spite of this, it is obvious that there is a considerable difference about cell attachment that could be related to the loaded cobalt ions. On the other hand, DAPI nuclear staining was carried out to detect cell density on the scaffolds and showed in Figure 7 at 1, 3 and 7 days after the cell culturing. These scaffolds were chemically crosslinked before the cell seeding by EDC/NHS. It is clear that the cell number was enhanced over time, although the cells were arranged on the composite scaffolds as a colony rather than a single model. These colonies could be related to the higher cell proliferation on the composite scaffolds compared to the naked scaffolds. It could be resulted that the higher cell confluence of the non-doped and doped composite scaffolds, depends on the lower contact angle and higher stiffness, respectively. In both reasons, the presence of 45s5 bioglass nanoparticles with or without cobalt ions is the main factor for the cell growth. However, the more cell number with the doped scaffold type, signifies the important role of cobalt ions. It is supposed that the higher stiffness with this scaffold enforced the surface rigidity and at last, the higher cell attachment and proliferation could be happened. The result is in the straight rout of the SEM examination, but not for the composite scaffold with the non-incorporated nanoparticles.

###  Scaffold biocompatibility by MTT assessment 

**Figure 8:**
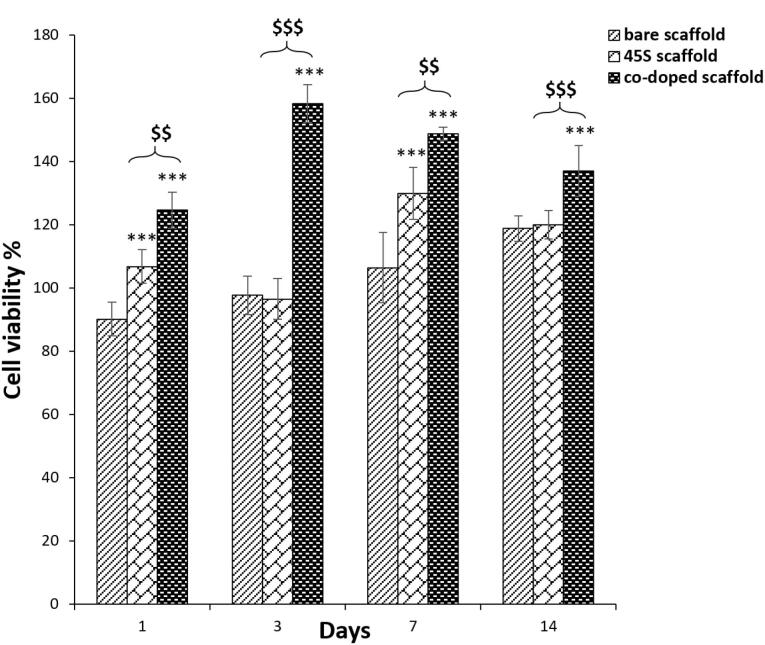
The cell viability percentages of the 2-layer bare, 3-layer 45s5 bioglass composite and cobalt doped-45s5 bioglass composite scaffolds after their crosslinking with EDC/NHS. The star signs indicate the difference between the bare and co-doped bioglass scaffolds and the dollar signs indicate difference between the non-doped and co-doped bioglass scaffolds

 Cellular growth rate on prepared scaffolds could be an important specification to find out a biocompatible substrate for tissue engineering. Thus, for this study, the cell survival was examined at 1st, 3rd, 7th and 14th days of the cell culture (Figure 8). Here, the relative growth ratios or cell viability percentages of the test groups were normalized by using the OD values of TCPS group. In contrast to the negative slope after 3 days, their cell viability values were more than 80%, representing the zero and first degree of toxicity. In accordance with before studies, these degrees are defined as a criterion for biocompatibility^89^ and this observation is verified for the all scaffold groups. It is better to be mentioned that the higher cell viability than 100% approved that substrate may have mitotic influence on cells.^90^ There are a few insignificant relations between the bare and 45s5 bioglass scaffold and regardless of some fluctuations, it could be resulted that there are no considerable differences between these groups (*P* > 0.05). Although, there was a sharp cell flattening by SEM images approving distinguishable surface rigidity between these scaffolds. In opposite, the significant higher value of the composite scaffold with cobalt ions is obvious (*P* < 0.0005) at the all predetermined time points. This higher cell viability of the corresponding group confirmed its better bioactivity after the bioglass nanoparticle coupling by cobalt ions. However, in accordance with SEM data about cell protrusion, the corresponding positive impact was expected. The all cell viability levels between the control and the cobalt bonded 45s5 scaffold showed statistically significant (*P* < 0.005 and 0.0005). The lower OD value of this group at 7 and 14 days compared to 1st and 3rd time points, could be related to the complete covering of the substrate surface by the cells. This cell density may make nutrient-starvation and then, the cell survival will be decreased.^91^ For dermal regeneration, there is emergency to employ the substrates with capability to enhance cell division as resulted for the composite scaffold with cobalt ions^92^ in the present assay.

###  Gene expression of L-929 fibroblasts by real-time PCR

**Figure 9:**
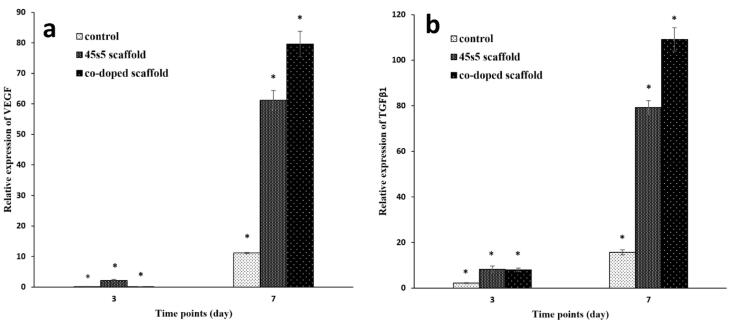
The results of real-time PCR method for the bare, 45s5 bioglass composite and cobalt doped-45s5 bioglass composite scaffolds after their crosslinking with EDC/NHS. The all groups were normalized against TCPS as the reference group. The star signs indicate the statistical differences between the scaffolds and TCPS

 The expression of marker genes is a most powerful method to insure about the efficiency of scaffolds for healing aims. For this assay, the all groups including the control and composite scaffolds were calibrated against TCPS group as a reference. This procedure is done to remove the impact of the differences about the amount of starting cDNAs. The housekeeping gene of GAPDH was utilized for the normalization step. The examined preferred genes were TGFβ1 and also, VEGF. The first one progresses angiogenesis and fibroplasia and makes collagen deposition to reorganize ECM.^93,94^ TGFβ1 is secreted by keratinocyte and fibroblasts after acute derma injuries.^95^ VEGF is one of the most important factors for dermal wound healing to stimulate angiogenesis and this biomolecule is generated by keratinocytes and fibroblasts.^96^ It was found that these cells could start to express this factor from the first day of cell inductions.^97^ Accordance with Figure 9a, the both non-doped and doped bioglass nanoparticles are effective on the expression of VEGF at the time points of 3 and 7 days. A study approved considerable VEGF expression after 72 hours by fibroblasts.^18^ Also, the gene evaluation by Real-Time PCR method determined that these nanoparticles can upregulated TGFβ1^98^ (Figure 9b). On the other hand, when cobalt ions are doped on bioglass nanoparticles, can activate VEGF expression.^99^ Although, there are some studies that investigated the positive role of cobalt ions on the TGFβ1 secretion,^100^ but there is no study about the cell treatments with the doped cobalt ions to bioglass. Except the first time point (3 days after), it seems that the cobalt ions have predominant signals on the VEGF expression on the 7^th^ days of the cell seeding and the relation between the scaffold with the intercalated and non-intercalated nanoparticles is significant (*P* < 0.05). Additionally, it can be concluded that the bare and 45s5 bioglass scaffolds are successful to some extent. However, this manner is negligible compared to the co-doped scaffold with the fold change value of 79.57 ± 4.2 specially against the control group. The results are more interested about TGFβ1 and again, the cobalt ions doped scaffold increased the expression to 109.13 ± 5.1. These considerable expression values of TGFβ1 and VEGF, makes the composite scaffold with the cobalt ions substituted bioglass nanoparticles as a candidate for dermal tissue engineering.

## Conclusion

 Herein, a scaffold with desired cell adhesion and spreading is considered to introduce a candidate for dermal tissue engineering. However, the possible movements of fibroblasts on these scaffolds were not studied in the present study and thus, this phenomenon would be evaluated in future researches. The 3-layered architecture of these scaffolds was fabricated to mimic the structure of normal skin tissue. On the other hand, due to some limitations about the employment of biological agents for dermal tissue engineering, 45s5 bioglass nanoparticles and also, their doped form by cobalt ions were concerned in this study. Accordance with the acceptable mechanical data, the polymer types of PCL and PU were selected correctly and also, by considering the cell culture outcomes, the crosslinked collagen by EDC/NHS facilitated cell attachments sufficiently. The composite scaffold with the doped nanoparticles had the highest value of young modulus confirming this scaffold as a stiff substrate. The corresponding stiffness was needed to induce cell adhesion strongly. This scaffold had lower elongation percentage compared to the composite scaffold with the non-doped nanoparticles. Although, its strain was ample to resistance against dermal stretching. The significant differences between the contact angle values of before and after chemical crosslinking, demonstrated that this process was done accurately. The higher contact angle of the both composite scaffolds depicted that the scaffold with the doped nanoparticles neutralized its biglass charges by cobalt ions strongly, but the scaffold with the non-bonded nanoparticles, preserved their charges. This residual electrostatic feature is needful to form ionic bonds with water molecules and reduces surface tension. However, with the values lower than 90°, these appropriate conditions for cell attachments are expected. Moreover, the fiber diameter of the scaffolds was kept same after the crosslinking indicating that the grafting method was not so acute to destroy fiber homogenously. In same manner, the fiber diameter of the composite scaffold was not altered after the doping of its nanoparticles with cobalt ions and therefore, these metals are not capable to increase the conductivity of the solution significantly. But it should be discussed that the degradation of the scaffold was increased considerably after the substitution of the bioglass nanoparticles with cobalt ions. This result could be correlated to the presence of higher free space between the scaffold polymer fibers in the latter scaffold and thus, the water diffusion is facilitated drastically. Also, the alkaline influence of metal ions must not irrelevant and the corresponding higher pH condition makes its faster degradation. The cell observations confirmed better cell spreading for the doped nanoparticle composite scaffold due to its higher stiffness. On the other hand, this scaffold had more cell density by DAPI staining and the lower degree of toxicity was obtained for this scaffold in compared to the other groups. Its zero-degree toxicity is interested rather than the first one of the other scaffold groups. Also, the Real-Time PCR assay approved the higher expression of TGFβ1 and VEGF for this scaffold predicting more ECM synthesis by using this scaffold. At the end, the scaffold with the doped nanoparticles would be taken as a more optimized scaffold to preserve the fibroblast ability to develop new skin tissue (Table 3). However, its antibacterial and also, fibroblast homing should be studied in future surveys.

## Competing Interests

 All authors declare they have no conflict of interests and also, no concerned financial issues that could have influenced on this article.

## Ethical Approval

 There are no ethical issues about this study due to no *in vivo* or clinical evaluations.
